# Restoration of Genuine Sensation and Proprioception of Individual Fingers Following Transradial Amputation with Targeted Sensory Reinnervation as a Mechanoneural Interface

**DOI:** 10.3390/jcm14020417

**Published:** 2025-01-10

**Authors:** Alexander Gardetto, Gernot R. Müller-Putz, Kyle R. Eberlin, Franco Bassetto, Diane J. Atkins, Mara Turri, Gerfried Peternell, Ortrun Neuper, Jennifer Ernst

**Affiliations:** 1 Division of Plastic, Aesthetic and Reconstructive Surgery with Hand Surgery, Brixsana Private Clinic, Julius Durst 28, 39042 Bressanone, Italy; 2 Clinic of Plastic, Reconstructive and Aesthetic Surgery, Padova University Hospital, Via Nicolo Giustiniani 2, 35128 Padova, Italy; franco.bassetto@unipd.it; 3 Institute of Neural Engineering, Graz University of Technology and BiotechMed Graz, Stremayrgasse 16/4, 8010 Graz, Austria; gernot.mueller@tugraz.at; 4 Division of Plastic and Reconstructive Surgery, Massachusetts General Hospital, Harvard Medical School, 55 Fruit Street, Boston, MA 02114, USA; keberlin@mgh.harvard.edu; 5 Department of Physical Medicine and Rehabilitation, Baylor College of Medicine, 7200 Cambridge St Ste 10C, Houston, TX 77030, USA; diane.atkins@bcm.edu; 6 Stroke Unit, Department of Neurology, Bolzano General Hospital, Lorenz-Böhler-Str. 5, 39100 Bolzano, Italy; turrimara@gmail.com; 7 Rehabilitation Clinic Tobelbad, Austrian Workers’ Compensation Board (AUVA), Doktor-Georg-Neubauer-Str. 6, 8144 Tobelbad, Austria; gerfried.peternell@auva.at; 8 Ludwig Boltzmann Institute for Traumatology, The Research Center in Cooperation with AUVA, Austrian Cluster for Tissue Regeneration, Donaueschingenstr. 13, 1200 Vienna, Austria; ortrun.neuper@trauma.lbg.ac.at; 9 Department of Trauma Surgery, Hannover Medical School, Carl-Neuberg-Str. 1, 30625 Hannover, Germany; jennifere@t-online.de

**Keywords:** targeted sensory reinnervation, phantom limb pain, neuropathic pain, phantom limb map, limb map, phantom hand, targeted muscle reinnervation, neuroma prophylaxis, pattern recognition, hand amputation, transradial amputation, sensory feedback, electroencephalogram

## Abstract

**Background/Objectives**: Tactile gnosis derives from the interplay between the hand’s tactile input and the memory systems of the brain. It is the prerequisite for complex hand functions. Impaired sensation leads to profound disability. Various invasive and non-invasive sensory substitution strategies for providing feedback from prostheses have been unsuccessful when translated to clinical practice, since they fail to match the feeling to genuine sensation of the somatosensory cortex. **Methods:** Herein, we describe a novel surgical technique for upper-limb-targeted sensory reinnervation (ulTSR) and report how single digital nerves selectively reinnervate the forearm skin and restore the spatial sensory capacity of single digits of the amputated hand in a case series of seven patients. We explore the interplay of the redirected residual digital nerves and the interpretation of sensory perception after reinnervation of the forearm skin in the somatosensory cortex by evaluating sensory nerve action potentials (SNAPs), somatosensory evoked potentials (SEPs), and amputation-associated pain qualities. **Results:** Digital nerves were rerouted and reliably reinnervated the forearm skin after hand amputation, leading to somatotopy and limb maps of the thumb and four individual fingers. SNAPs were obtained from the donor digital nerves after stimulating the recipient sensory nerves of the forearm. Matching SEPs were obtained after electrocutaneous stimulation of the reinnervated skin areas of the forearm where the thumb, index, and little fingers are perceived. Pain incidence was significantly reduced or even fully resolved. **Conclusions:** We propose that ulTSR can lead to higher acceptance of prosthetic hands and substantially reduce the incidence of phantom limb and neuroma pain. In addition, the spatial restoration of lost-hand sensing and the somatotopic reinnervation of the forearm skin may serve as a machine interface, allowing for genuine sensation and embodiment of the prosthetic hand without the need for complex neural coding adjustments.

## 1. Introduction

The hand, a magnificent sensory organ with highly developed sensory functions, enables an incredible spectrum of gross and fine motor movements. Immanuel Kant appropriately described the hand as the outer, visible part of the brain, as the tool of the mind. The hand’s capacity for sensation is fundamental to its complex functioning and is integral for social interactions. Hand loss is a life-changing event and affects the ability to move, work, interact with others, and maintain independence. Ongoing pain, painful phantom limb phenomena, and emotional trauma can complicate recovery. To date, the focus in the development of prostheses for the upper extremity has been on improving the control of myoelectric prostheses as well as the expansion of prehension patterns and mechanical resilience. With improved motor control of the prosthetic hand, restoring sensation, or sensory feedback, has now become a priority. “Tactile gnosis” describes the functional sensibility of the hand [1]. The interplay between peripheral function of the nerve and the central interpretation of sensory information in the somatosensory cortex and associated areas of the brain is important for successful adoption of prostheses [2]. Cutaneous receptors in the hand allow for discriminative touch, sensitivity to pressure, vibration, temperature, and stretching. The feedback system between the hand and the brain, with continuous proprioception and tactile input, is coordinated with the brain’s memory systems and is the prerequisite for the regulation of complex hand functions [3,4]. As a result, an insensate hand can lead to enormous disability. Discontinuation and resulting deafferentation and -efferentiation disrupt the neural pathway and can lead to functional reorganization of the somatosensory cortex. Presently, this is assumed to be the major mechanism for the development of neuroma pain (NP) and phantom limb pain (PLP) [5,6]. Targeted muscle reinnervation (TMR) and pattern recognition systems improve the motor control of prosthetic devices. Despite these advances, however, the rejection rate of the prosthesis remains very high. Thus, sensory feedback systems have been designed to restore sensation afforded by these high-tech devices as well as seeking to increase their usage and acceptance and to reduce injury-related pain. Various invasive and non-invasive sensory substitution strategies, including electro-, vibro-, and mechano-tactile, have been tested for their capacity to provide sensory feedback from a prosthesis but have so far been unsuccessful in translation to clinical practice [7,8,9]. One reason for this is the lack of matching the stimulus to physiologically natural, relevant, or genuine sensation—a key requirement for providing prostheses with sensory feedback [10,11]. As the available implantable electrodes for direct nerve stimulation still lack selectivity and stability, there have been attempts to directly bypass neural records by rerouting the sensation of the missing limb. TMR is a surgical approach in which the residual mixed nerve branches from the amputated hand are rerouted to reinnervate muscles at the residual limb (RL). The reinnervated muscles serve as additional control sites for myoelectric prostheses designed to improve functional outcomes as well as to reduce PLP [12]. After TMR, randomized reinnervation of the overlying skin was observed due to nonspecific redirection of the sensory afferents of the transposed nerves [13]. This observation seemed to be a promising neural approach, bypassing the need for implantable nerve interfaces while still allowing good spatial acuity of somatotopic sensation—touch, pain, temperature, and proprioception in the phantom limb—when the reinnervated skin is mechanically or electrically stimulated [13,14,15,16]. Fascicular targeted sensory reinnervation (TSR) describes a refined technique. To identify sensory fascicles of the median and ulnar nerve, Hebert et al. used intraoperative SEPs [17]. Those fascicles were separated and redirected to target cutaneous sensory areas of the intercostobrachial cutaneous and axillary cutaneous nerve branches in transhumeral amputees. This technique creates a discrete spatial sensory hand map over a selected area of receptor skin, allowing for an interface for sensory feedback and thus enabling the amputees to improve control of the myoelectric prostheses. Results showed effective recovery of discriminative pressure sensation of up to 85% accuracy [17,18,19].

Based on our experience using TSR on the lower limb [20], we here report on the development of a novel surgical method for upper-limb TSR (ulTSR) using similar principles. Here, we performed ulTSR in a case series of seven patients with different indications for hand amputation. With this technique, a limb map (LM) is created on the residual limb of the forearm, on which the sensory system of the amputated hand, including all fingers, can be drawn. The reinnervated skin and its resultant LM can provide an interface for (vibrotactile) feedback systems in such a way that the sense of touch can be passed on to the brain as a genuine feeling. Synchronized with the motion of a myoelectric hand prosthesis, stimuli can again reach the somatosensory area in the cortex that became reorganized following an amputation.

## 2. Materials and Methods

### 2.1. Patient Enrollment

After obtaining approval from the local ethics committees in Austria (EC Klagenfurt no. M2022-24) and Italy (EC Bolzano no. 50-2022), the clinical data of the first seven transradial amputated patients who underwent ulTSR at three medical centers from September 2020 to January 2024 were retrospectively analyzed. The three centers were high-volume hospitals with extensive experience in amputation surgery, and experienced surgeons performed the ulTSR. Table 1 lists the inclusion and exclusion criteria for ulTSR.

TSR was performed either as a therapeutic approach to treat PLP and/or NP in five patients or, at the time of the amputation, as prevention of PLP and NP in two patients. In total, we performed eight ulTSR operations on seven patients, including one bilateral case. All patients had different etiologies and indications for hand amputation. A summary of the patients is shown in Table 2.

All patients were thoroughly treated by a specialized pain physician and assessed by a psychologist before surgery. Prior to surgery, these patients were also subjected to a thorough assessment, including neurological and high-resolution ultrasound examinations, to evaluate the state of the existing neuroma and the presence of neuroma pain. In cases of uncertainty, consideration should also be given to advanced radiological techniques. Modern modalities such as 3D high-resolution ultrasound and magnetic resonance microscopy may prove valuable in distinguishing fascicular patterns, aiding in the detailed assessment of the affected nerve and guiding surgical planning [21].

### 2.2. Surgical Technique

**Figure 1:**
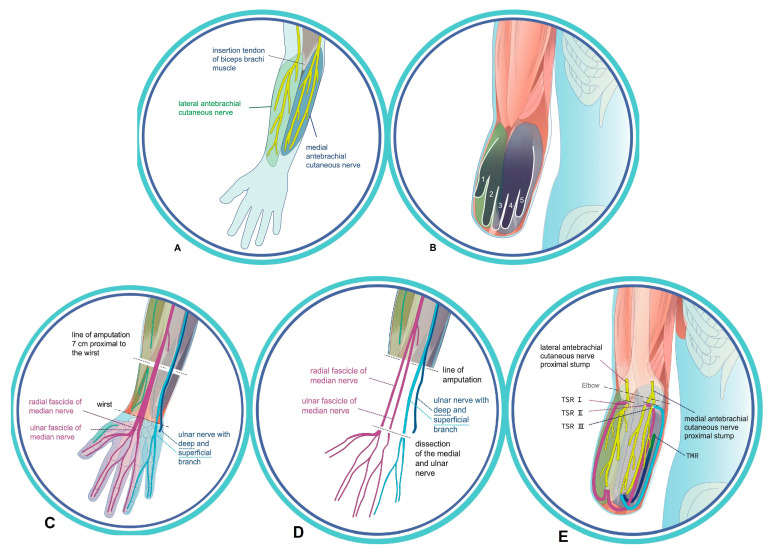
(**A**) Recipient nerves on the forearm; (**B**) LM (=phantom hand with fingers 1–5) after reinnervation. (**C**) Drawing of the amputation level and preparation of the median and ulnar nerves. (**D**) Microsurgical separation of the two fascicles of the median nerve and the two branches of the ulnar nerve. (**E**) Transposition of the separated two median nerve fascicles and two ulnar branches with performance of ulTSR I-III and TMR below the elbow joint.

All surgical procedures were performed by plastic surgeons with extensive experience in microsurgery and peripheral nerve surgery, with junior trainees assisting during the procedures. Depending on whether the TSR is performed at the same time as primary amputation of the hand, or secondarily, following the amputation, the skin incision is made on the palm of the hand or at the distal end of the residual limb. In principle, the sensory branches of the median and ulnar nerves must be exposed distally, separated from the motor branch, and dissected in order to perform end-to-end neurorrhaphy with the recipient sensory nerves of the forearm so that the patient becomes able to perceive their entire hand, including fingers (Figure 1A,B).

We performed the operation using general anesthesia, axillary plexus anesthesia, and a tourniquet. Amputation was planned 7 cm proximal to the wrist. After exposing the median and ulnar nerves (Figure 1C), both were separated microsurgically into their two fascicles and branches [22] (Figure 1D). Next, the medial and lateral antebrachial cutaneous nerves were exposed at the elbow. The branches of the median and ulnar nerves were tunneled subcutaneously to the proximal skin incisions below the elbow fold, followed by osteotomy of the ulna and radius 8–9 cm proximal to the wrist. To prevent neuroma of the superficial branch of the radial nerve and the dorsal branch of the ulnar nerve, an end-to-end neurorrhaphy with 9/0 nylon epineural single-button sutures was performed. Alternatively, regenerative peripheral nerve interfaces (RPNIs) could be performed at this point [23]. Once the nerves in the distal forearm had been addressed, the tourniquet was removed. Now the TSR procedure per se continued at the elbow, with a total of three microsurgical nerve coaptations enabling three TSR interfaces (Figure 1E):

TSR I: the radial fascicle of the median nerve (RFm) is connected end-to-end to the distal end of the lateral antebrachial cutaneous nerve by 9.0 epineural single-button nylon sutures.

TSR II: the ulnar fascicle of the median nerve (UFm) is coaptated end-to-end to the distal end of the lateral branch of the medial antebrachial cutaneous nerve by multiple 9.0 epineural single-button nylon sutures.

TSR III: the superficial branch of the ulnar nerve (SBu) is coaptated end-to-end with 9.0 single-button nylon epineural sutures to the distal end of the medial branch of the medial cutaneal antebrachial nerve. Finally, TMR of the deep branch of the ulnar nerve (DBu) was carried out, in which a motor branch of the superficial flexor muscle was selected and coaptated to the nerve (Table 3) (Video S1: Animation of the surgical technique).

**Figure 2:**
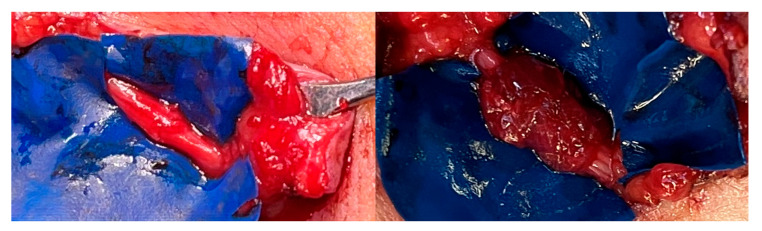
End-to-end re-coaptation and RPNI wrapped around the coaptation site as neuroma prevention.

For neuroma prevention at the three coaptation sites, RPNIs were wrapped around the coaptation site, harvesting an approximately 2 × 2 cm denervated muscle strip sutured with resorbable 5.0 single-button sutures [23]. Fibrin glue stabilized the construction (Figure 2).

### 2.3. Monitoring of Reinnervation Process and Rehabilitation Protocol

To protect healing of the wound and the nerve coaptations, wearing liners or sockets was prohibited for four weeks. TENS therapy (transcutaneous electrical nerve stimulation—manufacturer Chattanooga, model REHAB) was started after completion of wound healing (generally after two–three weeks) for 15 min twice a day, a total of 30 min, with an application of up to 500 joules. The overall rehabilitation was accompanied by standard integrative physio and occupational therapy, including sensory reeducation, grasping exercises, and training in the activities of daily living. Follow-up visits at baseline and at three, six, nine, and twelve months and then every six months thereafter were scheduled to monitor reinnervation, sensation capacity, and expected restoration of an LM. During these visits, clinical examination included percussion of the forearm according to the Hoffmann–Tinel sign. Sensory qualities such as pressure and temperature capacity were assessed by touching the forearm skin with hot (39° C) and cold (frozen) square packs. Reinnervation was monitored by percussing along the course of the coaptation site and expected innervation (Hoffmann–Tinel sign). Skin areas where the patients sensed their hand were assigned to an LM. The LMs were assessed longitudinally. Eventually, the patients drew LMs on their forearms by themselves during the observation time. The patients were also asked in an open, non-directed interview to describe their limb perceptions verbally. After the reinnervation reached the forearm skin and showed clear somatotopy of the thumb and fingers two to four, the patients were fitted with a custom-made vibrotactile feedback system, implemented in the existing socket of the prosthesis. As soon as the reinnervation was complete, a sensory glove prototype called Feelix (Saphenus Medical Technology GmbH, Vienna, Austria) was applied to the hand prosthesis as an add-on. Pressure on the fingertips was converted into vibrotactile stimulation, which transmits sensory perception and improves control and dexterity with the myoelectric hand prosthesis [9].

### 2.4. Sensory Nerve Action Potentials (SNAPs)

The sensory nerve action potentials (SNAPs) were recorded orthodromically using the near-nerve technique [24]. The sensory area of the medial antebrachial cutaneous nerve was stimulated through pre-gelled surface electrodes. Supramaximal stimuli were continuously delivered until reproducible SNAPs from ulnar and median nerves, at the elbow and cubital fossa, respectively, were recorded. The sensory area of the lateral antebrachial cutaneous nerve was stimulated with the same stimulation protocol until a reproducible SNAP from the median nerve was recorded. Averaging was used to identify small responses. SNAP protocol was repeated every three months after surgery.

### 2.5. Somatosensory Evoked Potentials (SEPs)

**Figure 3:**
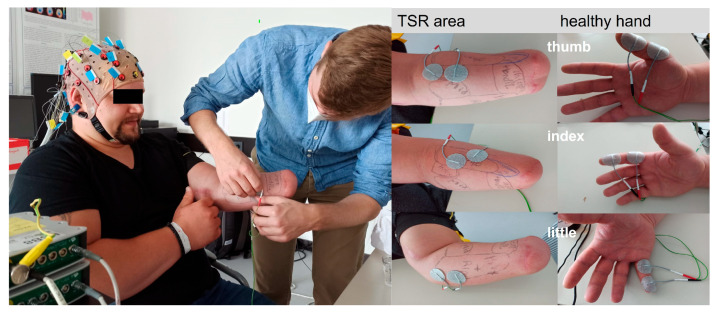
Experimental setup for SEP measurement. (**Left**) EEG cap attached and setup of electrodes at stimulation areas. (**Middle**) Electrode placement for stimulation of thumb, index, and little finger. (**Right**) Stimulation setup for thumb, index ,and little finger on the healthy hand.

Electrocutaneous stimulation was applied on the thumb, index, and little fingers of the intact hand. Similarly, electrocutaneous stimulation was applied to the reinnervated forearm skin areas where the thumb, index, and little finger were felt (Figure 3).

Every finger received 600 stimulations with biphasic, square electrical pulses of 300µs pulse width, with an interstimulus interval of 1 s. The amplitude of stimulation was adapted to each patient and area so as to elicit a clear sensation but no muscle twitch. EEG was recorded from 32 Ag/AgCl channels using two connected 16-channel EEG amplifiers (g.USBamp, g.tec Medical Engineering, Graz, Austria).

### 2.6. EEG Analysis

The 32-channel EEG signals were bandpass-filtered between 0.5 and 50 Hz using a two-way Least Squares FIR filter (eeglab). The HEAR algorithm was used to remove pop, drifts, and impulsive artifacts [25]. The EEG signals were then epoched into trials of −0.3 s and +0.7 s relative to the stimulation onset. An independent component analysis (ICA) was applied to remove physiological and non-physiological artifacts. SEPs were extracted by averaging over all trials and all three fingers. The procedure for removing ICA components involved minimizing the average amplitude of the central EEG channels (with the aim of increasing the magnitude of the N component of the SEP). To avoid any bias towards one hemisphere and ensure the detection of the most negative deflection, both left and right hemisphere channels were considered. This process was carried out sequentially, wherein one component at a time was removed. If the average amplitude decreased, then that particular component was identified for removal.

## 3. Results

### 3.1. Clinical Outcomes and Adverse Events

**Figure 4:**
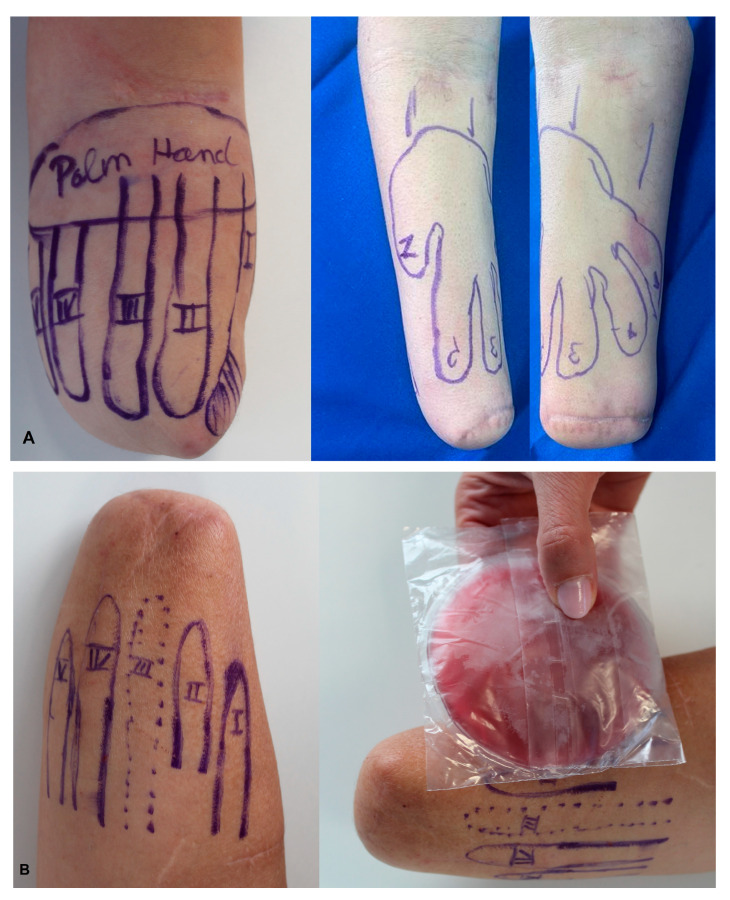
(**A**) Self-drawn LM by the patient is shown on the left forearm stump of patient 4 and on the right forearm of patient 6, both 5 months after undergoing ulTSR. For patient 6, the entire limb map is visible by rotating the forearm into a supinated position. (**B**) LM drawn by patient 3 on the right forearm 5 months after ulTSR. Perception of the ice pad as a cold sensation on the lateral edge of the LM corresponding to the thumb.

First, sensory reinnervation sings were observed after 1.5–2 months after TSR surgery and completed on average at six months. Reinnervation of the forearm skin gradually enabled patients to draw a LM of their amputated hand. Touching of the skin elicited genuine sensation with a discrimination capacity whose fineness ranged up to individual digits (Figure 4A).

No hypersensitivity within the LM could be detected in any of the patients. All patients were able to differentiate cold and warm sensations (Figure 4B).

Only patient 1 developed symptomatic neuromas (in continuity) at the coaptation sites, which required surgical revision. The neuromas were resected and the distal nerves re-coaptated. RPNIs were wrapped around the coaptation site for prevention of neuroma. The RPNI was subsequently added to the surgical routine (Figure 2). Since then, there has been no more symptomatic neuroma. For the five patients who had experienced preoperative pain, the pain either resolved completely or, in the case of patient 1, was extremely mild. NRS scores were significantly reduced. Notably, no particular pain medication was required in the long term. A summary of the clinical course is shown in Table 4.

### 3.2. Sensory Nerve Action Potentials (SNAPs)

Six months after TSR, in patient two the neurophysiological tests detected a small SNAP from the ulnar nerve by stimulating the sensory area of the medial antebrachial cutaneous nerve. Nine months after surgery, stimulation of the same area evoked a SNAP from the median nerve as well. One year after surgery, the stimulation of the sensory territory of the lateral antebrachial cutaneous nerve area also elicited a small SNAP from the median nerve. In the other six patients, the neurophysiological study six and twelve months after TSR detected reinnervation at all three surgical anastomoses. These are clear signs of sensory reinnervation.

### 3.3. EEG-Activity Patterns and Obtained Somatosensory Evoked Potentials (SEPs)

**Figure 5:**
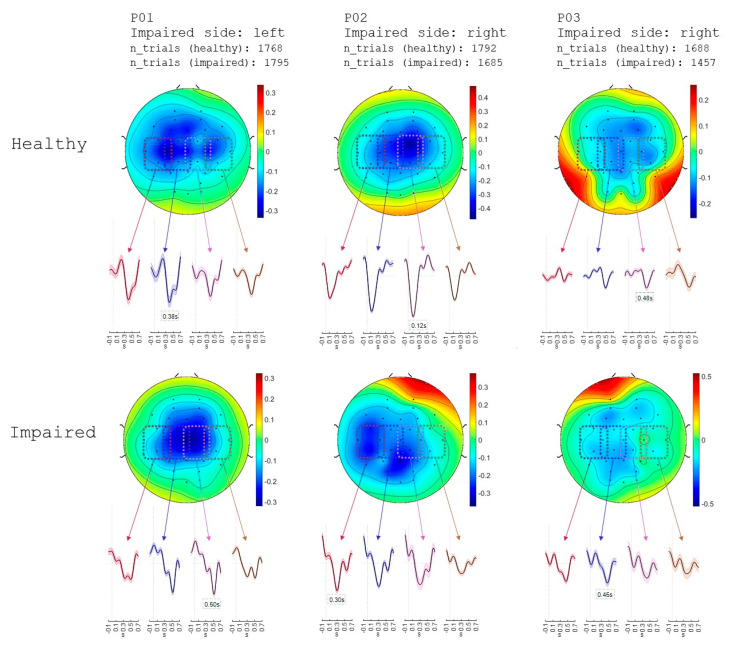
SEPs obtained from electrocutaneous stimulation applied on thumb, index and little fingers on the healthy hand (first row) and thumb, index and little finger area on the impaired side (second row) from three subjects (first column P01, second column P02 and third column P03). They are displayed after averaging groups of four channels as denoted by the colorcoded boxes on the topographical maps (red: FC5, CP5, C3, T7—blue: FC1, C3, CP1, Cz—purple: FC2, Cz, CP2, C4—brown: FC6, C4, CP6, T8). The topographical maps depict the spatial distribution of the electrical activity across the scalp at the time point of maximum negative SEP magnitude (denoted in textboxes within each subplot). The impaired side of each subject, as well as the number of trials used for averaging are shown on top of each subplot.

Figure 5 depicts the obtained SEPs from three subjects who had undergone an average number of trials ranging between 1457 and 1795. The topographical EEG maps revealed SEPs in the contralateral sensorimotor area for both healthy and impaired arms.

## 4. Discussion

Current approaches to restoring sensation have not succeeded in providing genuine sensation [26]; although, the phenomenon of afferent somatosensory nerve fibers that reinnervate the overlying dermis of a target muscle segment after a TMR procedure [13] was reported. At the time, subcutaneous defatting of that skin within TMR led to extensive sprouting of native sensory fibers into the skin, thereby triggering both native and referred cutaneous sensations [14]. Earlier TSR techniques enabled a widespread topographic representation of the digits with discrete separation of the median and ulnar hand maps in a defined targeted skin area, allowing for preservation of the somatotopy of the digits in transhumeral amputees [17,27]. This indicated that targeted reinnervation of the skin, following nerve redirection, provides a condition whereby afferents from the hand with an original high mechanoreceptor density and significant cortical representation can be displaced to a cutaneous surface [15,28].

Herein, we demonstrate the proof of concept of a new TSR technique, whereby a defined area of skin at the forearm residual limb is first denervated and then reinnervated by rerouted sensory nerves of the amputated hand and fingers. The advantage of this approach is that selective sensory nerve fibers—with the exception of the small number of motor fibers for the thenar muscle—reinnervate their original target. This ensures a homogeneous reinnervation of sensory fibers solely to sensory receptor nerves and might be the reason for a genuine perception of the amputated hand on the forearm residual limb (limb map).

The EEG activity patterns (SEPs) indeed indicate and, to a certain extent, objectively demonstrate that ulTSR enables an interface-promoting restoration of the somatotopy of single fingers and tactile gnosis after hand loss. Previously, it has been shown that stimulation of an amputee’s residual limb skin allowed for only non-somatotopic sensations. Devices based on this earlier technique, called sensory remapping, have been developed and tested [18,19,28,29]. As soon as two months after ulTSR, during the course of the reinnervation process, spatially separated reinnervation of the thumb, index, middle, ring, and little finger on the skin of the forearm could be observed. At that stage, patients could reliably distinguish different sensory qualities, such as pressure, warmth, and cold (Figure 4A,B). As objective indications for reinnervation, SNAPs could be obtained 6 months after ulTSR, evoking sensations of the donor nerve when the recipient sensory nerves of the forearm were stimulated. The spatial restoration of the hand sensation is probably one of the main differences between ulTSR and the randomized reinnervation of the overlying skin occurring after TMR. The spatial representation after ulTSR, leading to an LM, might play an important role in tactile gnosis, allowing for the functional sensation capacity of the prosthetic hand. The somatotopically reinnervated skin could serve as a mechano-neural interface that enables the amputee’s brain to genuinely feel the prosthetic hand in the future without the user having to relearn the fundamentals of hand sensation [27]. While most prior studies focused on functional outcomes of sensory restoration for closed-loop prostheses, there are few qualitative reports of reduction in PLP.

Sensory restoration paired with functional use of a prosthesis may have clinically meaningful effects on pain, embodiment and acceptance of the body image and prosthetic device [30]. Our experience on lower-limb TSR has already demonstrated a reduction in neuroma pain, possibly by providing a pathway for the regenerating nerve to grow through and reach the appropriate target rather than forming a painful neuroma. All patients reported their lost limb to be in a natural and comfortable position, since the palmar digital sensory nerves of the hand led to reliable somatotopy and limb maps of the thumb and its individual fingers. The restoration of sensomotory congruence might be a relevant mechanism for the observed reduction in PLP and might prevent its occurrence [20]. Through a sensory feedback system combined with a myoelectric hand prosthesis, stimuli can once again reach the somatosensory area in the cortex that is functionless and empty after an amputation. These stimuli can be genuinely transmitted to the brain, allowing it to believe that the hand is still present and receiving sensory feedback. As a result, herein, we observed that prior to ulTSR, existing PLP was resolved or, in the case of primary amputation, did not occur at all. In further studies, it will be necessary to evaluate whether the recovery process following ulTSR increases the acceptance and integration of the lost hand into the normal, healthy body schema (e.g., embodiment).

The TSR technique described herein does not rely on any implantable foreign bodies, such as electrodes or magnets, which carry the risk of dislocation, migration, or dysfunction. As a result, this technique increases biosafety and functionality. To prevent the occurrence of symptomatic neuromas in continuity at the coaptation site, we routinely wrap RPNIs around the coaptation site of both the donor and the recipient nerves. Following the procedure, there has been no recurrence of painful neuroma formation in any of our patients [26,31]. To our knowledge, this is the first case series to investigate the interplay between the peripheral sensory capacity of the redirected residual nerves of the amputated hand and the interpretation of sensory input in the somatosensory cortex. Due to the design of this intervention study and the inclusion criteria, ulTSR was performed on only a small number of patients, serving as their own controls. This inherently restricts the generalizability of the findings and highlights the need for further research. Future studies should aim to include larger, randomized cohorts to provide a more robust comparison, potentially employing alternative surgical techniques. Additionally, improved standardization of follow-up measurements will be critical to ensure consistency and reliability in evaluating outcomes over time.

## 5. Conclusions

In conclusion, the targeted end-to-end redirection of the hand’s sensory palmar nerves to the forearm’s sensory nerves (TSR) has demonstrated the potential to restore the sensory hand map and preserve proprioception of each individual finger of the lost hand in transradial amputees. When combined with a non-invasive vibrotactile feedback system connected to the tactile sensors of the hand prosthesis, this procedure facilitates the transfer of haptic sensations to a reinnervated skin area, ultimately enabling the perception of genuine functional sensitivity and potentially treating or preventing amputation-associated pain. This mechano-neural interface shows promise in advancing the restoration of tactile gnosis in individuals with limb loss.

## Figures and Tables

**Table 1:** Inclusion and exclusion criteria of upper limb targeted sensory reinnervation.

Inclusion Criteria	Exclusion Criteria
Treatment-resistant phantom and neuroma pain	Injured skin area for reinnervation at the forearm stump
Acute or elective programmed amputation	Volar or circumferent skin grafts at the forearm
Partial brachial plexus injury with preserved flexion and extension function of the elbow and sensitivity in the volar forearm. Completely non-functional hand	Total brachial plexus injury severe injured median or ulnar nerve

**Table 2:** Summary of patients and their pre-operative status. ulTSR (upper limb targeted sensory reinnervation).

Patient	Sex	Age at Injury (Year of Injury)	Mechanism of Injury and Indication	Side	No. of Surgeries	Age at Amputation (Year of Amputation)	Time Between Injury and Amputation	Type of Amputation	Phantom Limb and Neuroma Pain	Date of ulTSR Surgery
1	Male	25 (2015)	Severe contusion trauma	Left	5	30 (2020)	5 years	Elective	Yes	16 September 2020
2	Male	42 (2017)	Amputation with steel cable-replantation attempt	Right	3	43 (2018)	1 years	Elective	Yes	21 September 2020
3	Female	61 (2021)	Congenital vascular disorder	Right	4	61 (2021)	0 day	Elective	Yes	24 February 2021
4	Female	28 (2023)	Car accident- decollement injury	Left	1	28 (2023)	0 day	Acute	No	5 April 2023
5	Male	51 (2023)	Waterhouse Friderichsen Syndrome	Bilateral	1	51 (2023)	48 days	Elective	No	15 June 2023
6	Male	17 (2009)	Motorbike accident- partial brachial plexus lesion	Right	3	31 (2023)	14 years	Elective	Yes	8 August 2023
7	Female	39 (2020)	Amputation due to meat grinder	Right	1	39 (2020)	0 day	Acute	Yes	22 January 2024

**Table 3:** Description of the nerves with their associated connections at the elbow. TSR (targeted sensory reinnervation), TMR (targeted muscle reinnervation).

	Dissected Nerve	Division in the Hand and Finger		Connection Nerves
Wrist	Radial fascicles median nerve (RFm)	Nn. digitales palmares communes I-II → Nn. digitales palmares proprii 1–5	Elbow	Lateral antebrachial cutaneous nerve (TSR I)
	Ulnar fascicles median nerve (UFm)	N. digitalis communis III → Nn. digitalies palmares proprii 6 und 7		Lateral branch of medial antebrachial cutaneous nerve (TSR II)
	Superficial branch of the ulnar nerve (SBu)	N. digitalis communis IV (Ramus superficialis nervi ulnaris) → Nn. digitales palmares proprii 8–10		Medial branch of medial antebrachial cutaneous nerve (TSR III)
	Deep branch of the ulnar nerve (DBu)			Motor branch of the superficial flexor muscle (TMR)

**Table 4:** Summary of post-operative results. TSR (targeted sensory reinnervation) PLP (phantom limb pain), NP (neuroma pain).

Patient	Date of TSR Surgery	Signs of Sensation	Sensation on Reinnervated Skin Area	Complications (Time After Surgery)	PLP/NP NRS Pain Scale		Drugs (Treatment)	
					Before TSR	After TSR	Before TSR	After TSR
1	16 September 2020	2 months after surgery	Whole hand with all fingers	Neuroma at coaptation side (1 year)	10	1	Opioids, Anticonvulsants, Metamizole, Antidepressant	Acetaminophen on demand
2	21 September 2020	1.5 months after surgery	Whole hand with all fingers	No	8	0	Opioids, NSAID	None required
3	24 February 2021	1.5 months after surgery	Whole hand with all fingers	No	4	0	Due to the underlying disease: Opioids, NSAID, Metamizole	None required
4	5 April 2023	2 months after surgery	Whole hand with all fingers	No	X	0	Acute trauma	None required
5	15 June 2023	2 months after surgery	Whole hand with all fingers	individual small skin necroses due to the underlying disease—Waterhouse Friderichsen Syndrome (3 weeks)	X	0	Due to the underlying disease: Anticonvulsants, Metamizole, Antidepressant	None required
6	8 August 2023	1.5 months after surgery	Whole hand with all fingers	No	10	0	Cannabis, NSAID, Metamizole	None required
7	22 January 2024	1.5 months after surgery	Whole hand with all fingers	No	10	0	Anticonvulsant, NSAID	None required

## Data Availability

All relevant data included in the study are given in this manuscript and are available from the corresponding author on reasonable request.

## Supplementary Materials

The following supporting information can be downloaded at: https://www.mdpi.com/article/10.3390/jcm14020417/s1, Video S1: Animation of the surgical technique.
